# A Hybrid Structure of Piezoelectric Fibers and Soft Materials as a Smart Floatable Open-Water Wave Energy Converter

**DOI:** 10.3390/mi12101269

**Published:** 2021-10-18

**Authors:** Sina Baghbani Kordmahale, Jitae Do, Kuang-An Chang, Jun Kameoka

**Affiliations:** 1 Department of Electrical and Computer Engineering, Texas A&M University, College Station, TX 77853, USA; sina_baq.k@tamu.edu; 2 Department of Ocean Engineering, Texas A&M University, College Station, TX 77843, USA; jitaedo@tamu.edu (J.D.); kchang@civil.tamu.edu (K.-A.C.); 3 Department of Civil and Environmental Engineering, Texas A&M University, College Station, TX 77843, USA

**Keywords:** renewable energy, water wave energy conversion, macro-fiber composite, piezoelectric energy converter, soft material, environmentally benign

## Abstract

An open-water wave energy converter (OWEC) made of a new soft platform has been developed by combining piezoelectric macro-fiber composites (MFCs) and a low-cost elastomer. In the past decades, numerous types of water wave energy conversion platform have been developed and investigated, from buoys to overtopping devices. These harvesters mainly use electromagnetic-based generators, and they have faced challenges such as their enormous size, high deployment and maintenance costs, and negative effects on the environment. These problems hinder their practicality and competitiveness. In this paper, a soft open-water wave energy converter is introduced which integrates piezoelectric MFCs and bubble wrap into an elastomer sheet. The performance of the OWEC was investigated in a wave flume as a floatable structure. The maximum 29.7 µW energy harvested from the small OWEC represents a promising energy conversion performance at low frequencies (<2 Hz). The elastomer was able to protect the MFCs and internal electrical connections without any degradation during the experiment. In addition, the OWEC is a foldable structure, which can reduce the deployment costs in real-world applications. The combination of no maintenance, low fabrication cost, low deployment cost, and moderate energy harvesting capability may advance the OWEC platform to its real-world applications.

## 1. Introduction

Renewable energy is a highly demanded instrument for economic and social growth, supply infrastructures in remote areas, and climate-change mitigation [1,2,3]. However, there are roadblocks to the development and investment in renewable energy-based technologies associated with policymaking, lack of financial incentive, mainstream energy firms’ interests, and a lack of skilled specialists in the field [4,5,6]. Nevertheless, growth in the use of renewable energy sources has become increasingly important in the current high-energy-consumption world. The main attractions of renewable energy sources are that they harvest energy from nature with fewer environmental impacts and have positive effects on the generating countries’ GDPs [6,7]. For instance, using solar energy to power remote mines and oil extraction facilities, or to provide electricity for remote sensing are two examples of the use of renewable energy in the modern era. Specifically, these are examples where renewable energy is provided for applications located far from a power distribution network, which can reduce the negative environmental effects [5,8,9,10].

An appropriate strategy for using renewable energy sources can be chosen based on the distribution of renewable energy sources on the planet and the energy demand [11]. The abundance of vibrational kinetic energy on earth makes its conversion to electricity for use in modern applications particularly attractive [12,13,14]. Water wave energy is one of the most abundant sources that can be used as a source of sustainable vibration energy [15]. To harvest electrical energy from water waves, four different transducers have been investigated: electromagnetic, electrostatic, triboelectric, and piezoelectric transducers [16,17,18,19].

Electromagnetic-based energy-harvesting platforms have some advantages, such as low impedance, and are suitable for large-scale energy production. Electromagnetic-based transducers in their conventional form need a resonance mechanical system to provide proper actuation of the magnet and coil in low-frequency conditions. To harvest energy from low-frequency vibrations, these harvesters rely on a frequency up-conversion mechanism. Resonance-based mechanical systems involve a high level of complexity and high design costs, as well as bulky structures, and high maintenance costs. As such, these mechanical structures and their negative features are the main drawbacks of using electromagnetic transducers for large-scale energy conversion. For small-scale energy harvesting, the fabrication of coils and magnets for micro-scale devices is expensive and difficult, which is another drawback of this conversion method [20]. Electrostatic-based mechanical energy converters apply charged variable capacitors. Their compatibility with microelectromechanical systems (MEMS) technology makes them suitable for small-scale energy conversion. The main drawbacks of this type of harvesters are their low power output and their dependency on an external voltage supply or electrets. Another main problem of electret-based devices is the low durability of charge in electrets [20,21]. 

Triboelectric-based harvesters use two mechanisms simultaneously: the triboelectric effect and electrostatic induction. The main issues with this complicated mechanism are the use of nano-structures in triboelectric nano-generators, complex design requirements, complications in finding the right pairs of materials, high levels of wear and tear, and low durability, alongside complexities associated with packaging and isolation [22,23]. Piezoelectric-based energy harvesters have an issue with regard to material limitations [24]. However, the main advantages of piezoelectric-based devices are the simple nature of piezoelectric transducers, which enable designers to use a simple mechanism in the harvester, in addition to their durability, high voltage, high impedance of output, and high energy production density [25,26]. Some vibrational energy converters use a combination of the aforementioned transducers to reach a higher conversion efficiency [27].

To increase electrical power generation using piezoelectric transducers, various techniques have been investigated such as force focusing and increasing mechanics [28], resonance up-conversion mechanics [29], using different piezo materials [30], using more efficient nonlinear electronics [31], and adjusting the resonance frequency to make a broadband energy converter [32]. All these techniques have drawbacks, such as the use of complex mechanics, the limitations of the materials, and the need for bulky buoys that make the whole energy converter complicated and increase the cost of maintenance and deployment.

Based on the required scale of energy and operation environment, the most optimal power generation system must be selected [33,34,35,36]. For this study, we integrated piezoelectric transducers and soft materials to produce OWECs and successfully deployed and tested them in a wave flume. The commercially available macro-fiber composites (MFCs) in the proper configuration, along with bubble wrap and encapsulation in the Ecoflex 030 biocompatible soft polymeric material, formed an OWEC with a flexible structure with no mechanical complexities and minimum environmental negative effects [37]. Fabricated OWECs, with varying numbers of MFCs were placed horizontally and their power performance was investigated in a floating configuration in the wave flume.

## 2. Design and Fabrication

A flexible floating structure made from biofriendly soft materials and containing flexible piezoelectric transducers is a good option to act as an OWEC with minimum negative environmental effects, and with an efficient and cost-effective design. Piezoelectric transducers have a high energy density production capability, which makes them an alluring option for this purpose [38], as they can help reduce the size of the OWEC and minimize the deployment costs. Moreover, to reduce the environmental drawbacks of conventional OWECs, an appropriate structural shape and environmentally friendly materials were selected to avoid acoustic noise impacts [39,40,41]. A further advantage of the piezoelectric conversion mechanism is that it can reduce the number of additional moving parts and consequent acoustic noise, as well as lowering maintenance costs [41,42,43].

The appropriate piezoelectric transducer must be matched with a flexible structure. MFC units can provide high energy density and a flexible structure when used as a piezoelectric transducer [44]. M17007-P2, which is manufactured by Smart-materials, was chosen for use in the proposed OWEC. This P2-type MFC uses the d_31_ piezoelectric index, which is smaller than the d_33_, for PZT fibers. While d_31_<d_33_, the aluminum-based bottom–top electrode topology that is used in P2-type MFCs increases the electric charge gathering and output current [45]. The PZT fibers were sandwiched between electrodes and Kapton films using epoxy. The average shear modulus of MFCs is 5.515 GPa, and the average capacitance is 98.5 nF for this model [46].

**Figure 1:**
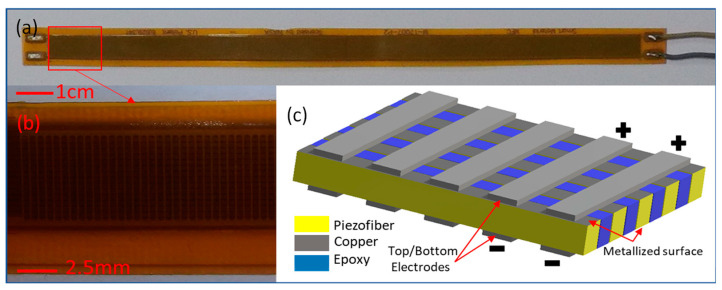
The proposed open-water wave energy converter (OWEC) with P2-type macro-fiber composites (MFCs), M17007-P2 model. (**a**) The P2-type MFC module using the d_31_ piezo index. (**b**) A magnified image of the MFC. The top electrode and the fibers can be seen clearly. (**c**) Schematic details of MFC: the bottom–top electrodes, together with top/bottom surface metallization of the piezo-fibers, form the internal connections of the MFC. The bottom–top electrodes’ configuration helps P2-type MFCs to collect more charge in comparison with P1-type MFCs, which have interdigitated electrodes.

The MFC and its structure is presented in Figure 1. Figure 1a depicts the photographic image of M17007-P2 MFC. Figure 1b is a magnification of the area marked by a red square box in Figure 1a; the electrodes are shown in this image. Figure 1c shows the 3D schematic diagram of the M17007-P2 MFC. The bottom–top electrodes of P2-type MFCs are depicted in this schematic diagram.

Ecoflex 030 with a shear modulus of 22.081 KPa has been chosen as an encapsulation platform because of its light, flexible, and cost-effective properties and resistance to highly corrosive saltwater [47]. Moreover, it is possible to shape this elastomer by using a soft-material casting method, which is cost-effective in comparison to other methods like standard lithography [48,49,50]. The mold for this purpose can be fabricated by a 3D printer [51]. To guarantee the buoyancy of the structure, a piece of bubble wrap was integrated into the Ecoflex as a flexible filler that reduces the overall density of the structure. Using these materials in a sheet-like form can create a floatable, bendable, and durable structure. This structure can then be used as an ideal platform and integrated with the proper piezoelectric elements to form the desired OWEC.

**Figure 2:**
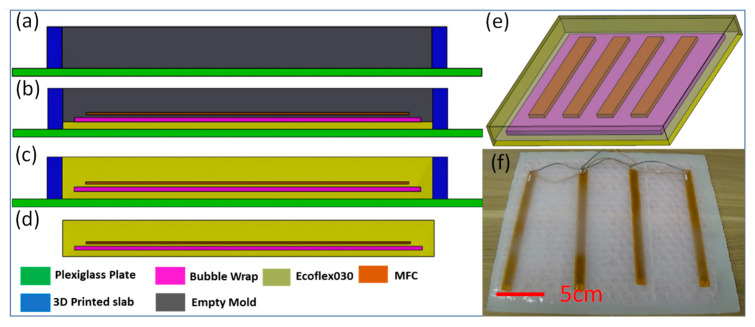
Fabrication steps, schematic, and photographic image of the OWEC (figure not to scale). (**a**) The empty mold. (**b**) A thin layer of Ecoflex was dispensed in the mold and the bubble wrap and MFC were placed on top of this layer. (**c**) A further layer of Ecoflex was dispensed over the MFCs and bubble wrap, and the mold was filled. (**d**) The Ecoflex was cured and the OWEC finalized. (**e**) 3D schematics of the OWEC, showing the bubble wrap and the MFCs. (**f**) Photographic image of the final OWEC.

Figure 2 presents the schematic diagram of the fabrication process, as shown previously [51]. Figure 2a depicts the schematic diagram of the empty mold. This mold is made by bonding individually fabricated 3D-printed parts onto a Plexiglas plate. As a first step, liquid Ecoflex 030 was dispensed on this mold until it was half-filled. Bubble wrap and MFCs were placed on the top of the Ecoflex 030 in succession, as depicted in Figure 2b. To finalize the fabrication of the OWEC, another portion of Ecoflex 030 solution was dispensed over these elements, as depicted in Figure 2c. After curing the Ecoflex for four hours at 25 °C, the elastomer was crosslinked, and the OWEC fabrication process was completed. The drawings for the final product are depicted in Figure 2d,e. Figure 2f presents a photographic image of an OWEC with four MFCs and dimensions of 250 mm × 250 mm × 4 mm. In terms of circuitry, a parallel connection configuration was used to maximize the output power of the OWECs used in this study, as described in the literature [45,52].

## 3. Results

### 3.1. Rectifier Circuit and Electrical Measurement

**Figure 3:**
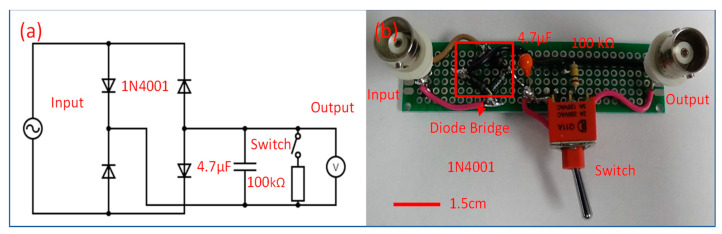
Schematic diagram of the rectifier circuit and photograph of the real circuit. (**a**) Schematic diagram of the rectifier circuit with the values of the elements. (**b**) Photograph of the real circuit.

We connected the OWECs with 2, 3, 5, and 10 MFCs to a diode bridge rectifier circuit, in order to investigate their performance in a wave flume under various wave conditions. The rectifier circuit was used to convert the OWECs’ AC output to DC. The DC output voltage and power were measured and analyzed. The schematic diagram and a photograph of the circuit are shown in Figure 3. The diode bridge was formed from 4X1N4001 diodes, and the tantalum capacitor had a capacitance of 4.7 µF [53]. To measure the open-circuit voltage, a Texas Instrument Data Acquisition system (DAQ) was connected directly to the rectifier circuit.

The time required for charging the capacitor was one minute. This was therefore the effective duration of each test. To measure the power delivered to the load, a nominal 100 K Ω resistor with an actual value of 99.35 K Ω was switched into the circuit as a resistive load. Power was determined using Equation (1).

(1) P=V2R

### 3.2. Wave Flume and Experimental Setup

**Figure 4:**
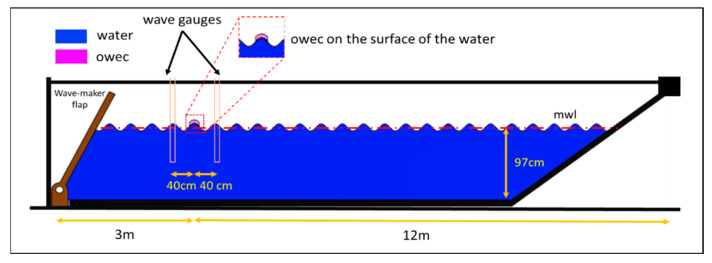
The schematic diagram of the wave flume and experimental setup (not to scale). OWECs were placed at a distance of 3 m from the wave maker. The total length of the flume was L = 15 m, and its width was W = 0.6 m. The height of the water was adjusted to H = 0.97 m during the experiments (L = 15 m × W = 0.6 m × H = 0.97 m). The wave gauges are also depicted.

A wave flume was used to perform experiments and test the buoyancy, electrical performance, power production, and stability of the OWEC developed for this study. The wave flume is equipped with a wave-maker flap that can produce waves with different periods and heights. The total length of the flume was L = 15 m and its width was W = 0.6 m. The depth of the water in the flume was fixed at H = 0.97 m during the experiments (L = 15 m × W = 0.6 m × H = 0.97 m). The OWECs were placed in the flume, anchored or unanchored, at a 3m distance from the wave maker. Wave gauges were placed in front and behind the center of the OWECs at a distance of 40 cm to measure the wave parameters. The experimental setup is presented in Figure 4.

During the experiments, two cameras were used to record all the details from the side and bird’s eye view.

### 3.3. Horizontal Test Conditions

**Figure 5:**
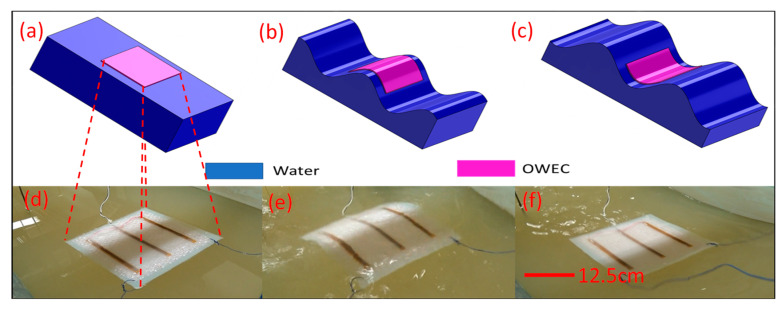
The wave energy converter and its mechanical behavior (schematics not to scale). (**a**–**c**) Schematics of the OWEC on the water surface following the surface profile of the water waves. (**d**–**f**) Photographic images of the OWEC on the water surface and its responses to the wave motion, following the surface profile.

The OWECs were placed horizontally on the surface of the water in the wave flume, as shown in Figure 5. In this floating configuration, the OWECs were moored to the walls of the flume by means of suspended ropes to avoid drifting as a result of the waves. Figure 5 presents a simplified drawing of the wave energy converter, its qualitative response to the wave, and photographic images of an actual floating device. Figure 5a–c depict schematic diagrams of a horizontally floating OWEC in the wave flume in still water conditions, on the convex surface of the wave, and on the concave surface of the water, respectively. Figure 5d–f present the photographic images of these same conditions, in the same order.

**Figure 6:**
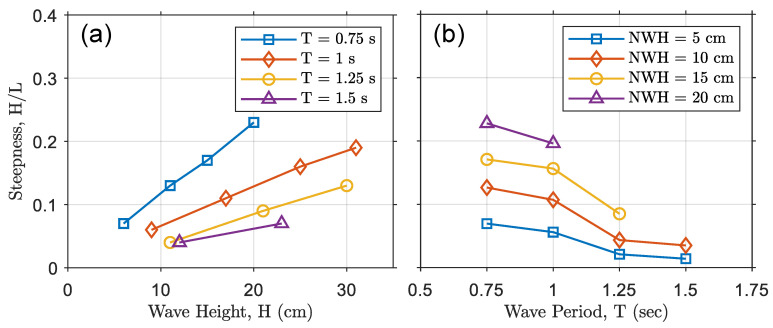
Wave steepness. (**a**) Variation in steepness versus wave height for a given wave period. (**b**) Variation in steepness versus wave period for a measured wave height.

In the flume tests, the tested wave periods of the monochromatic waves were 0.75, 1, 1.25, and 1.5 s while the nominal wave heights for each period were 20, 15, 10, and 5 centimeters. The real and nominal wave conditions are detailed in Table 1. This table also depicts the steepness of each wave condition (the ratio of the wave height, H, to the wavelength, L). In Figure 5, the steepness variation of the incoming waves is shown for each wave period and each nominal wave height. Figure 6a shows that the steepness of the wave was increased by increasing the wave height for each wave period, whereas Figure 6b shows that the steepness was decreased by increasing the wave period. Some wave conditions were avoided in the experiment, as shown in Figure 6 and Table 1, to prevent waves from breaking in the flume. Breaking waves can cause unwanted harmonics and produce standing waves in the flume, resulting in varying wave heights and frequencies, which can contaminate the results and invalidate tests.

### 3.4. Results and Discussion of Horizontal Test Conditions

The open-circuit voltage (V_OC_) and the power delivered to the resistive load (P_OUT_) are analyzed in this section. We investigated the output power against various experimental parameters, such as the number of MFCs inside the OWECs, the wave period, the wave height, and the wave steepness in the flume, as discussed in [55]. The output voltage and power from MFCs combined in parallel are proportional to their bending angle, speed, and acceleration. The shear modulus of Ecoflex 030 and M17007-P2 MFC are 22.081 kPa and 5.515 GPa, respectively. The stiffness of the OWECs varied according to the number of integrated MFCs, which is a critical parameter for determining the vibration modes and bendability of an OWEC. Due to these factors, there is always a trade-off between the bendability of the OWEC and its usefulness for energy harvesting. Given that the OWECs in this study follow the surface profile of the waves, higher output voltage and power can be expected from steeper waves.

**Figure 7:**
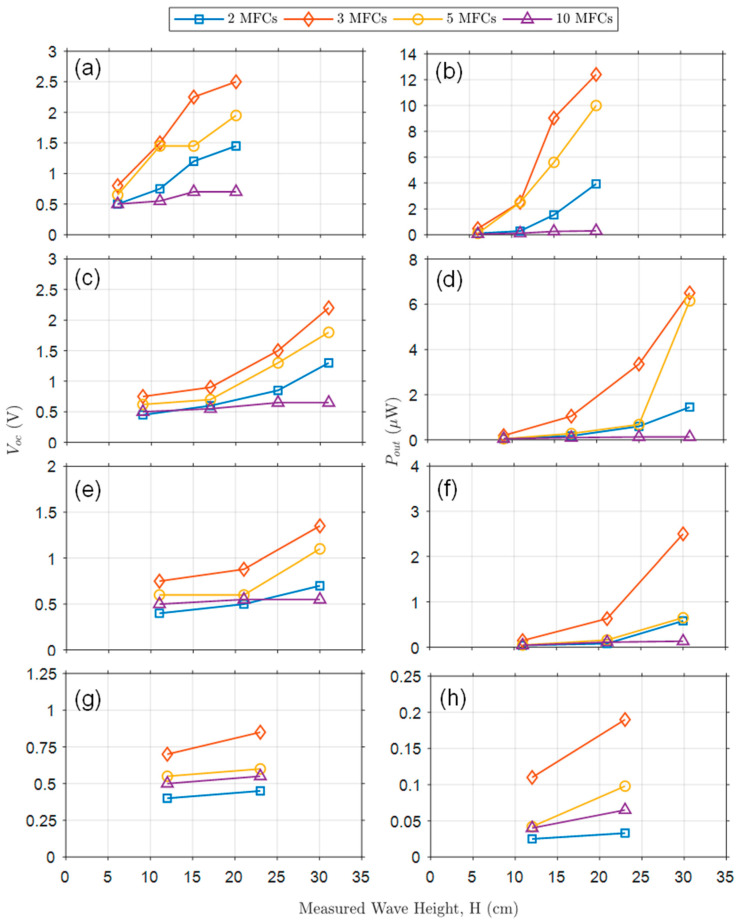
Graphs showing V_OC_ and P_OUT_ as a function wave height for a specific wave period and for OWECs with varying numbers of MFCs (2, 3, 5, and 10). (**a**,**b**) V_OC_ and P_OUT_ for 0.75 s wave period. (**c**,**d**) V_OC_ and P_OUT_ for 1.00 s wave period. (**e**,**f**) V_OC_ and P_OUT_ for 1.25 s wave period. (**g**,**h**) V_OC_ and P_OUT_ for 1.50 s wave period.

In Figure 7, changes in V_OC_ and P_OUT_ are shown as a function of varying wave heights at a specific wave period, for OWECs with varying numbers of MFCs. The test conditions represented in each graph are as follows: Figure 7a,b show V_OC_ and P_OUT_ with a wave period of 0.75 s and wave heights of 5, 10, 15, and 20 cm. Figure 7c,d show V_OC_ and P_OUT_ with a wave period of 1.00 s wave period and wave heights of 5, 10, 15, and 20 cm wave heights. Figure 7e,f show V_OC_ and P_OUT_ with a wave period of 1.25 s and wave heights of 5, 10, and 15 cm. Figure 7g,h show V_OC_ and P_OUT_ with a wave period of 1.50 s and wave heights of 5 and 10 cm. As shown in Figure 6a, increasing the wave height for a given wave period increases the steepness of the wave. V_OC_ and P_OUT_ follow the same pattern when they are measured for a specific wave period and incremental wave heights. Due to the increment in wave steepness, steeper waves can bend the OWEC more, producing a higher level of output voltage and power. However, it was found that a larger number of MFCs did not produce higher V_OC_ or P_OUT_ due to the resultant decrease in bendability of the OWEC. As shown in Figure 7, an OWEC with 3 MFCs was found to be optimal in the tests.

**Figure 8:**
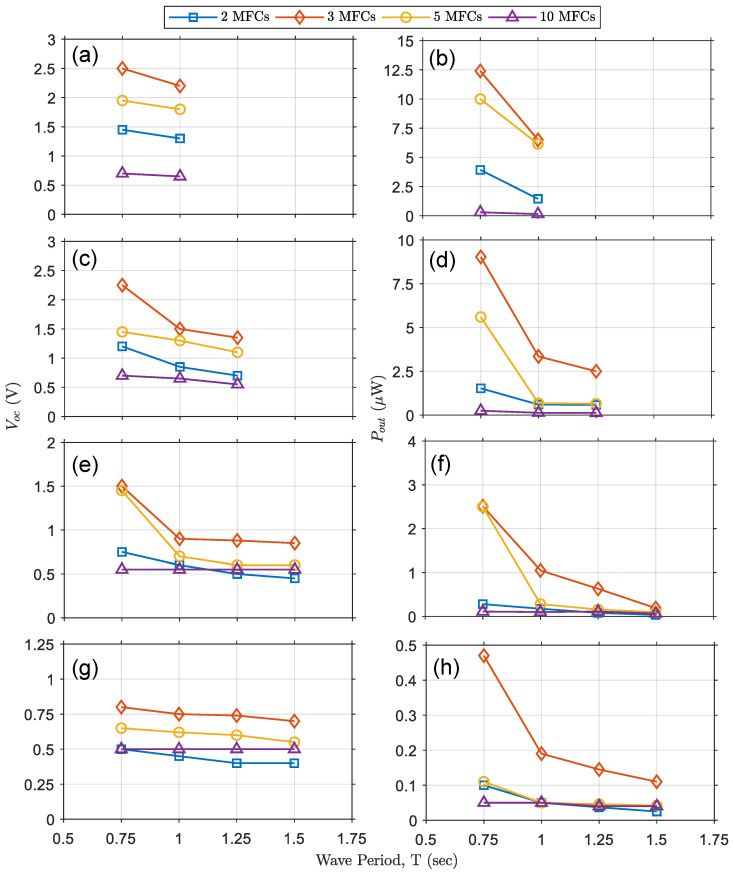
Graphs depicting V_OC_ and P_OUT_ as functions of wave period for a specific wave height and for OWECs with varying numbers of MFCs (2, 3, 5, and 10). (**a**,**b**) Output voltage and power for 20 cm nominal wave height and 0.75 and 1.00 s wave periods. (**c**,**d**) Output voltage and power for 15 cm nominal wave height and 0.75, 1.00, and 1.25 s wave periods. (**e**,**f**) Output voltage and power for 10 cm nominal wave height and 0.75, 1.00, 1.25 and 1.50 s wave periods. (**g**,**h**) Output voltage and power for 5 cm nominal wave height and 0.75, 1.00, 1.25 and 1.50 s wave periods.

In Figure 8, the variations in V_OC_ or P_OUT_ are depicted as a function of varying wave periods at a specific wave height. Similarly to Figure 7, results for OWECs with varying numbers of MFCs are shown on each graph. The test conditions represented in each graph are as follows: Figure 8a,b show V_OC_ and P_OUT_ with a wave height of 20 cm and wave periods of 0.75 and 1.00 s. Figure 8c,d show V_OC_ and P_OUT_ with a wave height of 15 cm and wave periods of 0.75, 1.00, and 1.25 s. Figure 8e,f show V_OC_ and P_OUT_ with a wave height of 10 cm and wave periods of 0.75, 1.00, 1.25, and 1.50 s. Finally, Figure 8g,h show V_OC_ and P_OUT_ with a wave height of 5 cm and wave periods of 0.75, 1, 1.25, and 1.5 s. As shown in Figure 6b, increasing the wave period lowers the steepness of the wave decreased, and this is reflected in decrements in V_OC_ and P_OUT_, as seen in Figure 8.

**Figure 9:**
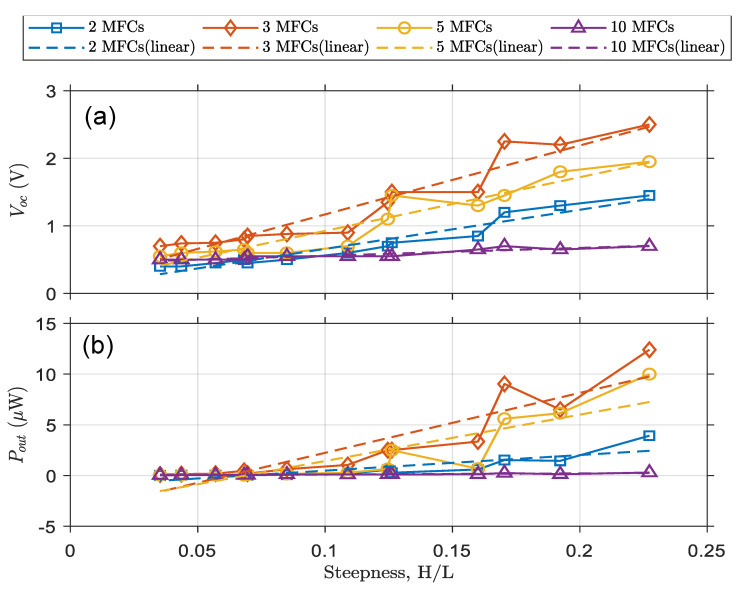
V_OC_ and P_OUT_ versus wave steepness for a OWECs with varying numbers of MFCs. (**a**) Output voltage versus wave steepness (**b**) Output power versus wave steepness.

In Figure 9, the open-circuit voltages and output powers are shown as functions of the wave steepness. In Figure 9a, the V_OC_ changes are depicted against waves of varying steepness. In Figure 9b, the P_OUT_ variations versus steepness are shown. The outputs increase almost monotonically when the wave steepness is increased. The distraction of monolithic behavior of outputs could be due to nonlinear effects that shifted the output values slightly. From the results shown in Figure 7, Figure 8 and Figure 9, it can be concluded that OWECs with 3 MFCs have the produce the highest power output under all the tested wave conditions. The maximum power output is a compromise between the OWEC’s bendability and power production. While OWECs with five and ten MFCs have a higher capacity to produce electrical energy, they have a stiffer structure with lower bendability. 

The proper encapsulation of the OWECs is crucial to ensuring their performance. As these electrical devices need to work in highly corrosive saltwater and under sunshine, the electrical components must be appropriately protected to endure the harsh environment. Each MFC has a capacitance of 93–100 nF. The number of MFCs, their internal connections, and the wiring determine the level of capacitance in a given OWEC. The capacitances achieved with the OWECs designed for this experiment were 190, 270, 470, and 850 nF for those with 2, 3, 5, and 10 MFCs, respectively. The capacitance was measured before, during, and after each experiment, and the results showed no observable variation. These persistent results point to the stable encapsulation of the MFCs in the soft-material platform. The output voltage and power also showed no deterioration, which suggests the high quality of encapsulation and the efficiency of the design. During the tests, the OWECs were exposed to saltwater for 336 hours and tolerated more than 41,000 cycles without showing any negative effect.

### 3.5. One-Sided Anchored Configuration

**Figure 10:**
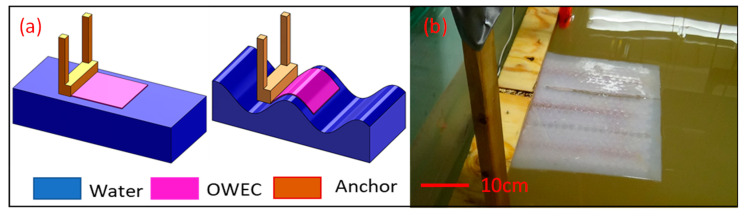
The horizontal one-sided anchoring (schematics not to scale). (**a**) Schematic of the one-sided anchored OWEC. (**b**) Photographic image of the one-sided anchored OWEC.

We found that OWECs with 3 MFCs demonstrated the highest power production ability. To study the effects of anchoring the OWEC on energy production, OWECs with 3 MFCs were anchored as shown in Figure 10.

**Figure 11:**
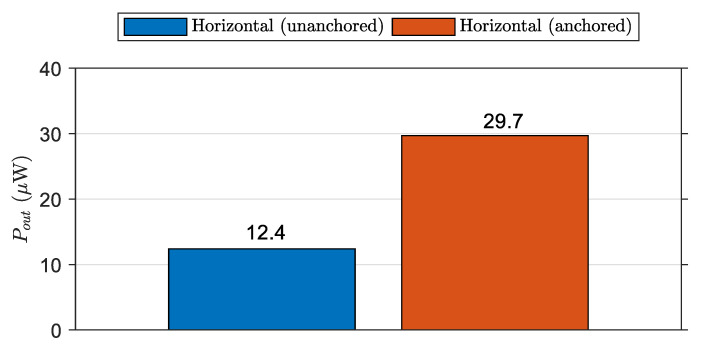
Comparison of unanchored free-floating OWEC and horizontally one-sided anchored OWEC. The OWEC can harvest substantially more energy in the anchored configuration.

The power production was measured under the conditions of a 20 cm wave height and 0.75 s wave period. As shown in Figure 7, the OWEC with 3 MFCs under these test conditions produced the maximum energy in the free-floating (horizontal unanchored) configuration. Figure 11 depicts the results of the energy production of OWEC with 3 MFCs in both free-floating (horizontally unanchored) and horizontally one-sided anchored configurations. From Figure 11, it can be seen that the maximum output power for the free-floating configuration was 12.4 µW. For the one-side anchoring configuration, the power production was more than twice that, at 29.7 µW. This implies that the one-sided anchoring method substantially increases the energy-harvesting ability of the OWEC. This one-sided anchor configuration may be especially useful in applications designed to supply energy to remote sensing equipment on offshore structures, as the OWECs can be anchored to these structures in a similar manner to the configuration shown in Figure 10.

## 4. Discussion

This study investigated the use of low-cost and environmentally friendly OWECs to harvest energy under various wave conditions in a flume. Commercially available MFCs and bubble wrap were employed and a low-cost casting method was used to fabricate OWEC devices incorporating various numbers of MFCs, used as piezoelectric conversion elements, encapsulated in Ecoflex 030 elastomer. Since the OWECs designed for this study have no internal moving parts, their maintenance and fabrication costs are significantly lower in comparison to other types of OWEC that contain complicated mechanics [42]. Furthermore, these OWECs are lightweight and foldable, which can potentially reduce their deployment costs [43] in a comparison with OWECs that require bulky buoys and wave attenuators [56].

The energy outputs from OWECs with 2, 3, 5, and 10 MFCs were investigated in a horizontally floating configuration. The OWEC with 3 MFCs demonstrated the highest wave energy conversion in this configuration (12.1 µW peak); the harvester containing 10 MFCs demonstrated the lowest output (0.3 µW peak). The energy harvester with 3 MFC was further investigated under a horizontally one-sided anchoring configuration and showed a promising 29.7 µW energy harvesting ability, equal to 0.52 mW/m^2^ and 12.9 mW/m^3^ if converted to energy per unit area and volume. Zhang et al. produced an OWEC based on TENG, which achieved a maximum power production of 54 µW, equal to 180.6 µW/m^2^ and 651 µW/m^3^ [57]. Rodrigues et al. achieved a maximum power output of 230 µW by placing four of their TENG modules on a 1:8 scale commercial buoy, equivalent to less than 2.26 mW/m^2^ and 6.02 mW/m^3^ if converted to energy per unit area and volume [58]. An energy harvester based on an electrostatic capacitive transducer, developed by Pruvost et al., produced a power output of 1.7 µW, equal to 0.24 µW/cm^2^ and 4.53 µW/m^3^ [59]. Taking into account the dimensions of these OWECs, the power generation from the OWEC developed in this study is more efficient than or comparable to those of in the above studies. The output voltage and power production remained consistent throughout the 336-hour experimental period. The capacitance of each OWEC remained stable before, during, and after the experiment, as did the internal electrical connections. This shows the consistent performance of the soft material-based encapsulation used to protect electrical elements from saltwater. Thanks to the low cost and the effectiveness of our approach, other types of transducers, such as clusters of electrostatic MEMS [60] or piezoelectric MEMS [61] transducers can also be integrated into the soft encapsulation. Similarly, this soft encapsulation approach can pave the way to using a network of triboelectric nanogenerators in the same floatable and foldable platform [62]. For real-life applications, several progressive paths can be considered. For instance, the OWEC introduced in this study can be used in its current form as a wave monitoring and remote sensing platform by placing it on offshore structures or mooring on the surface of open-water. The harvested energy can be used to drive sensors and for communication purposes, or the OWEC can be a self-energized sensor itself.

For large-scale energy harvesting, this proposed scalable OWEC could be fabricated to larger dimensions, with the same thickness but a much larger surface area. One of the limitations of scaling up the proposed OWEC is the limited size of commercially available MFCs, which are limited by the length of piezo-fibers as well as fabrication limitations. Nevertheless, integrating a higher number of small commercial MFCs into a bigger sheet of soft material and connecting them in an appropriate circuit configuration could still be a feasible method to scale up the OWEC’s energy-harvesting capacity. The deployment costs can be greatly reduced using such an OWEC because its bendability means it can be folded and then deployed. While the information reported in this article may be useful for the effective scaling up of OWECs with suitable stiffness, bendability, and power production capacity, further study should be undertaken to design more efficient OWECs for larger-scale energy production.

## Figures and Tables

**Table 1:** The nominal and measured parameters of waves. Water depth was fixed at H = 97 cm throughout the tests. (a) The wave period was determined by the period of the paddle in the wave flume. (b) Nominal wave heights were chosen and entered as the required heights to the LabVIEW program. (c) The measured wave height, H, is the real wave height, measured by the wave probes. (d) Wavelength, L, was determined by the wave dispersion relationship [54]. (e) The wave steepness (H/L) was the quotient of measured wave height to wavelength. “NA” indicates instances where the wave parameter was not well defined due to wave breaking and the subsequent generation of harmonics.

(a) Wave Period, T (s)	(b) Nominal Wave Height (cm)	(c) Measured Wave Height, H (cm)	(d) Wavelength L (cm)	(e) Wave Steepness (H/L)
0.75	20	20	88	0.23
0.75	15	15	88	0.17
0.75	10	11	88	0.13
0.75	5	6	88	0.07
1.0	20	31	156	0.19
1.0	15	25	156	0.16
1.0	10	17	156	0.11
1.0	5	9	156	0.06
1.25	20	NA	NA	NA
1.25	15	30	241	0.13
1.25	10	21	241	0.09
1.25	5	11	241	0.04
1.5	20	NA	NA	NA
1.5	15	NA	NA	NA
1.5	10	23	335	0.07
1.5	5	12	335	0.04
